# A Comparison of the Sodium Content of Supermarket Private-Label and Branded Foods in Australia

**DOI:** 10.3390/nu7085321

**Published:** 2015-08-21

**Authors:** Helen Trevena, Bruce Neal, Elizabeth Dunford, Hila Haskelberg, Jason H. Y. Wu

**Affiliations:** 1 The George Institute for Global Health, Sydney Medical School, University of Sydney, P.O. Box M201 Missenden Road, Camperdown, Sydney, NSW 2050, Australia; E-Mails: bneal@georgeinstitute.org.au (B.N.); edunford@georgeinstitute.org.au (E.D.); hhaskelberg@georgeinstitute.org.au (H.H.); jwu1@georgeinstitute.org.au (J.H.Y.W.); 2 The School of Public Health, Faculty of Medicine, Epidemiology and Biostatistics, Imperial College of Science, Technology and Medicine, Praed Street, Norfolk Place, London W2 1PG, UK; 3 Royal Prince Alfred Hospital, Missenden Road, Camperdown, Sydney, NSW 2050, Australia

**Keywords:** sodium reduction, pre-packaged food, branded, private-label

## Abstract

Supermarket private-label products are perceived to be lower quality than their branded counterparts. Excess dietary sodium in foods contributes to high blood pressure and cardiovascular disease. Sodium concentrations in products are an important indicator of quality. We compared the sodium content of 15,680 supermarket private-label and branded products, available in four Australian supermarkets between 2011–2013, overall and for 15 food categories. Mean sodium values were compared for: (1) all products in 2013; (2) products in both 2011 and 2013; and (3) products only in 2013. Comparisons were made using paired and unpaired *t* tests. In each year the proportion of supermarket private-label products was 31%–32%, with overall mean sodium content 17% (12%–23%) lower than branded products in 2013 (*p* ≤ 0.001). For products available in both 2011 and 2013 there was a ≤2% (1%–3%) mean sodium reduction overall with no difference in reformulation between supermarket private-label and branded products (*p* = 0.73). New supermarket private-label products in 2013 were 11% lower in sodium than their branded counterparts (*p* = 0.02). Supermarket private-label products performed generally better than branded in terms of their sodium content. Lower sodium intake translates into lower blood pressure; some supermarket private-label products may be a good option for Australians needing to limit their sodium intake.

## 1. Introduction

While there remains some debate [1] excess dietary sodium has been identified by the World Health Organization (WHO) as a modifiable risk factor for raised blood pressure and a major contributor to cardiovascular disease (CVD) [2]. In Australia, 11% of deaths from ischemic heart disease and 15% of deaths caused by stroke are attributable to excess dietary sodium [3]. The estimated average salt intake of Australian adults is 9 g/day (3500 mg sodium/day) [4,5] more than twice the Australian government’s Suggested Dietary Target of 4 g/day (1600 mg sodium) [6]. As 75% of dietary sodium is contributed by packaged processed foods [7] reducing added sodium in these foods would be a cost effective strategy to reduce the burden of preventable CVD [8,9,10,11].

In Australia the supermarket sector is the largest retail industry [12] and the majority of packaged processed food items are bought at supermarkets [13]. For millions of consumers, supermarkets offer significant convenience as they provide a one-stop-shop made possible by the wide range of food categories stocked [14,15]. The packaged food items within each food category may be branded and owned by the supermarket, and sold exclusively in the supermarkets’ own stores. Such items are often referred to as “supermarket brands”, “own label” or “home-brand”, and are hereafter referred to as “private-label” products. Supermarkets may also retail “branded products”, which are items owned by national and international food manufacturers and distributed to the general trade [16]. While very few studies have conducted in-depth analyses comparing the nutritional quality of private-label *versus* branded products, consumers have traditionally perceived private-label products to be of lower quality than their branded counterparts [16,17,18]. Some retailers differentiate their private-label offering between value-, mid- and premium products [16]. Likewise, the dollar share of private-label sales differs tremendously between countries (0%–45%) and between product categories, with total share predicted to rise in Australia from 24% to nearer 30% [16,18,19,20,21,22].

Due to their large market share and popularity with consumers, supermarkets have substantial power to influence the healthfulness of the food environment by determining what products get onto the supermarket shelf. Supermarket demands to lower added sodium in their private-label products could be a significant contributor towards Australian efforts to meet the voluntary global target of a 30% reduction in the mean population intake of sodium by 2025 [23]. Three of the four largest Australian supermarkets (ALDI, Coles and Woolworths have made voluntary commitments to reduce sodium content across nine food categories as part of the Australian Food and Health Dialogue (FHD) initiative which was launched in 2009 [24]. Prior analyses suggest modest progress in sodium reduction for three food categories [25] but whether the pace of change differed between private-label and branded products’ was not assessed. The objective of this study was to compare the sodium content of private-label *versus* branded products across a broad range of food categories available in Australian supermarkets. Products were examined in both 2011 and 2013 to allow evaluation of changes over time.

## 2. Methods

### 2.1. Data Collection

Between 2011 and 2013, data were collected in the fourth quarter of each year from the same four supermarkets (ALDI, Coles, IGA/Metcash, and Woolworths) in Sydney, Australia. Data were obtained directly from the mandatory Nutrition Information Panel (NIP) on product packaging but where exactly the same branded product was for sale in more than one supermarket, it was recorded only once. Likewise, where the same private-label or branded product was presented in different pack sizes only one entry was recorded. For each product, the manufacturer, brand and product name, as well as the nutritional information per 100 g were recorded. Where the brand of the product was a proprietary brand name of the supermarket it was considered as a private-label product of that supermarket. Data were entered into The George Institute’s branded food composition database [26] according to standardised procedures [27]. Data were verified according to a defined quality assurance protocol and workflow which included screening for outliers and missing values, checking of data entry accuracy by two study personnel and resolving queries and discrepancies by a review of the original NIP data, consultation between the research personnel, review of the manufacturer website or follow-up with the manufacturer directly. Ethics approval was not required.

### 2.2. Identification of Food Categories

Major food categories included were those typically containing added sodium. In addition the category was required to contain at least 20 private-label and 20 branded products in each year to allow meaningful comparison and statistical inference. Categories included were biscuits; bread; breakfast cereals; cakes, pastries and muffins; cereal bars; cheese; crisps and snacks; desserts; nuts and seeds; processed fish; processed meat; ready meals; sauces; soup; and, vegetables. Table S1 lists the major food categories reported, foods included, and the number and percentage of private-label products in 2013.

### 2.3. Products Excluded

Products were excluded where the brand and manufacturer name could not be identified from information on the pack and we were unable to confidently confirm whether it was a private-label or branded product.

### 2.4. Outcomes

The primary outcome for the study was the mean sodium content (mg/100 g) determined from data reported on the NIP. The mean value was calculated by summing the sodium values in mg/100 g (assuming the density of liquid products was 100 g/100 mL) across included products and dividing through by the number of products.

### 2.5. Statistical Analysis

The mean, median and range of sodium content were first calculated overall and for each food category. Since the sample sizes were sufficiently large to not require assumptions of normality, analyses and reporting are based upon mean values and parametric tests [28]. There were four main analyses done:

(1) Comparison of the mean sodium values of private-label *versus* branded products for all products available for sale in 2013 (*n* = 5995). Differences in mean sodium content between private-label and branded foods were determined and compared using unpaired *t* tests.
(2) Comparison of the mean sodium values of private-label *versus* branded products for the subset of products available for sale in both 2011 and 2013 (*n* = 2792). Changes in mean sodium between 2011 and 2013 were assessed by paired *t* tests.
(3) Comparison of the mean sodium values of private-label *versus* branded products for the subset of products first introduced to the market in 2013 (*n* = 1870), differences in means were assessed using unpaired *t* tests.
(4) Comparison of the mean sodium content of private-label products for each of the four supermarkets with data plotted graphically for 2011, 2012 and 2013 and unpaired *t* tests used to compare the 2013 mean values for all products combined.

In each case the analyses were done for all product categories combined and separately for the 15 major food categories studied. The primary analysis excluded data for 186 products in five minor sub-categories of products which had extreme sodium values and are consumed in small quantities (canned herring; capers; peppers/capsicum and other picked vegetables; satay and curry pastes; and black-bean/Asian ambient sauces) out of a total of 214 minor sub-categories. Sensitivity analyses were also done with these products included. Statistical significance was defined as two-sided α = 0.05. Formal adjustments for the multiplicity of testing were not made. However, all findings were interpreted in light of the number of comparisons made, the practical significance of any differences observed, and with a focus on the primary outcomes. Analyses were conducted using Stata 13.1 (Stata Corp., College Station, TX, USA).

## 3. Results

### 3.1. Products Identified for Private-Label and Branded Categories

Between 2011 and 2013 NIP data for 15,680 products was recorded for the 15 major food categories analysed; 2011 (*n* = 4501), 2012 (*n* = 5184), 2013 (*n* = 5995). The total number of products in each category for all years ranged from 374 for desserts, to 2156 for sauces. Four private-label suppliers were included—ALDI, Coles, IGA (Metcash) and Woolworths. In 2013, the food category with the lowest percentage of private-label products was sauces (*n* = 100, 17%) and the highest was cakes, pastries and muffins (*n* = 196, 52%). However, for the majority of food categories (60%) the proportion of private-label products in a category ranged from 25% to 35% (Table S1). The overall proportion of private-label products was stable (31%–32%) across the three years. Likewise, for each food category, there was little variation between the years (±6% difference) with the exception of breakfast cereals in which the proportion of private-label products increased from (*n* = 32, 19%) in 2011 to (*n* = 85, 31%) in 2013 (Table S2).

### 3.2. Mean Sodium Content of all Private-Label and Branded Products Available in 2013

The mean sodium content of all private-label products was 17% lower compared to branded products (−90 mg/100 g, 95% confidence interval −119 to −62; *p* < 0.001) (Table 1). Assessed by food category, the mean sodium content of private-label products was lower compared to branded products for desserts by 27% (−30, −58 to −1 mg/100 g), biscuits by 24% (−110, −151 to −67 mg/100 g), processed meats by 22% (−245, −321 to −168 mg/100 g) and breads by 7% (−32, −56 to −9 mg/100 g) (all *p* < 0.04). The opposite was true for private-label breakfast cereal products which had a 37% higher mean sodium content (+53, +4 to +100 mg/100 g; *p =* 0.03). Mean sodium content did not differ significantly between private-label and branded products in 2013 for any of the other 10 categories.Results were similar in sensitivity analyses that included the five minor food subcategories with items present with extreme sodium values, and private-label having 26% lower mean sodium (−156, −188, to −123 mg/100 g; *p* ≤ 0.001, Table S3).

**Table 1:** Mean sodium levels (mg/100 g) in all supermarket private label and branded products on the supermarket shelves in 2013 and the differences between them, overall and for 15 major food categories.

Food Category	Supply Type	*n* (%)	Mean Sodium mg/100 g ± SD	Mean Difference (Private Label—Branded) mg/100 g (95% CI)	*p*-Value ^1^
All products	Branded	4146 (69)	527 ± 655	−90 (−119, −62)	<0.001
	Private label	1849 (31)	437 ± 454		
Biscuits	Branded	631 (75)	450 ± 344	−110 (−151, −67)	<0.001
	Private label	214 (25)	340 ± 242		
Bread	Branded	178 (64)	453 ± 100	−32 (−56, −9)	0.01
	Private label	99 (36)	421 ± 85		
Breakfast cereals	Branded	191 (69)	144 ± 160	+53 (+4, +100)	0.03
	Private label	85 (31)	197 ± 193		
Cakes, muffins, pastries	Branded	181 (48)	308 ± 138	−16 (−45, +12)	0.26
	Private label	196 (52)	291 ± 145		
Cereal bars	Branded	137 (75)	137 ± 101	+14 (−20, +49)	0.43
	Private label	46 (25)	151 ± 110		
Cheese	Branded	393 (73)	752 ± 371	−49 (−120, +22)	0.17
	Private label	145 (27)	703 ± 372		
Crisps and snacks	Branded	200 (65)	664 ± 419	+14 (−77, +105)	0.76
	Private label	109 (35)	678 ± 326		
Desserts	Branded	96 (73)	113 ±109	−30 (−58, −1)	0.04
	Private label	36 (27)	83 ± 55		
Nuts and seeds	Branded	198 (62)	118 ± 238	+22 (−32, +76)	0.42
	Private label	120 (38)	140 ± 234		
Processed fish ^2^	Branded	287 (63)	395 ± 149	−24 (−52, +5)	0.10
	Private label	169 (37)	371 ± 152		
Processed meat	Branded	336 (65)	1095 ± 491	−245 (−321, −168)	<0.001
	Private label	179 (35)	850 ± 375		
Ready meals	Branded	172 (67)	295 ± 140	−23 (−11, +58)	0.18
	Private label	84 (33)	318 ± 108		
Sauces ^2^	Branded	489 (83)	1032 ± 1430	−82 (−383, +219)	0.59
	Private label	100 (17)	950 ± 1219		
Soup	Branded	193 (76)	281 ± 68	−14 (−29, +10)	0.31
	Private label	60 (24)	271 ± 63		
Vegetables ^2^	Branded	464 (69)	359 ± 456	−51 (−124, +21)	0.16
	Private label	207 (31)	308 ± 408		

^1^ *p*-Value derived from unpaired *t*-tests, *p* ≤ 0.05 for a difference in sodium content between private label and branded grouped products; ^2^ Food groups excluded from Sauces (satay ambient sauces, curry pastes, black bean/Asian ambient sauces); vegetables (capers, peppers/capsicum, other pickled vegetables); processed fish (canned herring). For comparison between all supermarket private label and branded products see Table S1.

### 3.3. Change in Mean Sodium Content between 2011 and 2013

**Figure 1:**
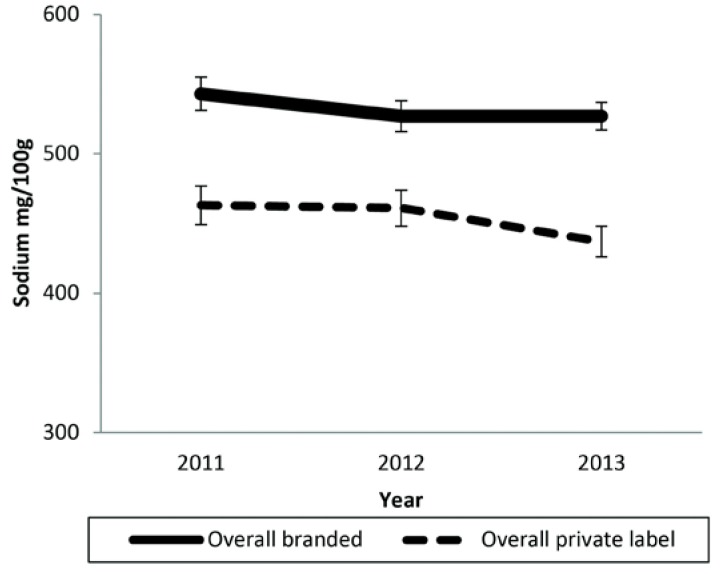
Mean sodium content (mg/100 g) and SE of overall private-label and branded products (2011, 2012, 2013).

The overall mean sodium content for private-label products was 463 mg/100 g in 2011 (*n* = 1434), and 437 mg/100 g (*n* = 1849) in 2013 representing a non-significant 6% mean reduction in sodium concentration between 2011 and 2013 (−26, −60 to +8 mg/100 g; *p* = 0.13) (Figure 1; Table S2). Likewise, there was a non-significant 3% mean reduction in sodium concentration for branded products (−15, −47 to +16 mg/100 g; *p* = 0.34). In each of the three years mean sodium of private-label products was between 13% and 17% lower than branded products (all *p* ≤ 0.001). Private-label biscuits (all *p* ≤ 0.001) and processed meat (all *p* ≤ 0.03) had a lower mean sodium content compared to branded products in all three years but the findings varied for other categories (not shown).

### 3.4. Changes in Mean Sodium for Reformulated Products

To evaluate the extent of product reformulation we studied the 2792 products that were available in both 2011 and 2013 (Table 2). There were small (≤2%) but statistically significant (both *p* < 0.001) reductions in the overall mean sodium concentration of both private-label and branded products between 2011 and 2013 but no evidence to suggest greater reformulation to lower sodium content in private-label products (*p* = 0.73). At the food category level significant reductions in mean sodium were observed for private-label products in two categories and for branded products in seven categories, but in every case the sodium reductions were less than 10%. For bread products there was borderline significant evidence (*p* < 0.04) that reformulation of private-label products was greater than for branded products. The proportion of products for which there was no change in sodium content for private-label (*n* = 578/808) and branded (*n* = 1411/1984) was almost identical at 72% and 71%, respectively.

**Table 2:** Mean sodium levels (mg/100 g) in supermarket private label and branded products on the supermarket shelves in both 2011 and 2013 and the differences between them over those three years, overall and for 15 major food categories.

	Supply Type	*n*	Year		Difference within Private Label or within Branded (2013–2011)		Difference between Private Label and Branded (2013–2011)	
			2011	2013				
			Mean Sodium mg/100 g ± SD	Mean Sodium mg/100 g ± SD	Mean Change mg/100 g (95% CI)	*p*-Value ^1^	mg/100 g (95% CI)	*p*-Value ^2^
All products	Branded	1984	551 ± 721	544 ± 725	−7 (−13, −3)	<0.001	−2 (−9, +7)	0.73
	Private label	808	439 ± 449	430 ± 442	−9 (−15, −4)	<0.001		
Biscuits	Branded	253	473 ± 340	464 ± 328	−9 (−17, −2)	0.01	+8 (−4, +22)	0.19
	Private label	95	332 ± 225	331 ± 221	−1 (−11, +9)	0.87		
Bread	Branded	89	473 ± 93	460 ± 95	−13 (−21, −5)	<0.01	−17 (−35, −1)	0.04
	Private label	35	458 ± 78	427 ± 73	−31 (−49, −13)	0.02		
Breakfast cereals	Branded	97	180 ± 188	164 ± 169	−16 (−23, −9)	<0.001	+10 (−6, 26)	0.23
	Private label	21	185 ± 214	179 ± 207	−6 (−17, +5)	0.27		
Cakes, muffins, pastries	Branded	56	377 ± 111	373 ± 114	−4 (−18, +8)	0.46	+1 (−17, 19)	0.87
	Private label	53	325 ± 116	322 ± 122	−3 (−16, +10)	0.60		
Cereal bars	Branded	50	145 ± 100	142 ± 100	−3 (−7, +2)	0.30	+3 (−7, +13)	0.61
	Private label	12	163 ± 114	163 ± 114	0 (0, 0)	0		
Cheese	Branded	185	763 ± 373	770 ± 370	+7 (−7, +20)	0.33	−28 (−56, 0)	0.05
	Private label	66	721 ± 379	700 ± 369	−21 (−49, +6)	0.13		
Crisps and snacks	Branded	104	715 ± 385	656 ± 373	−59 (−88, −30)	<0.001	+18 (−1, +67)	0.47
	Private label	50	650 ± 333	609 ± 313	−41 (−79, −3)	0.04		
Desserts	Branded	40	55 ± 28	56 ± 27	+1 (−2, +3)	0.65	+1 (−6, +8)	0.72
	Private label	14	51 ± 18	53 ± 24	+2 (−8, +12)	0.68		
Nuts and seeds	Branded	104	101 ± 252	101 ± 252	0 (<−0.01, +0.03)	0.32	+1 (−5, +7)	0.70
	Private label	71	172 ± 235	173 ± 239	+1 (−6, +8)	0.74		
Processed fish	Branded	165	405 ± 134	405 ± 133	0 (−11, +11)	1.00	−1 (−17, +15)	0.89
	Private label	109	376 ± 146	375 ± 147	−1 (−11, +8)	0.81		
Processed meats	Branded	131	1050 ±496	1029 ±483	−21 (−42, −1)	0.04	−1 (−39, +37)	0.96
	Private label	65	895 ± 397	873 ± 362	−22 (−58, +13)	0.21		
Ready meals	Branded	51	263 ± 55	256 ± 55	−7 (−13, −1)	0.01	+10 (−2, +23)	0.10
	Private label	21	275 ± 64	278 ± 58	+3 (−11, +17)	0.65		
Sauces	Branded	283	1091 ± 1514	1088 ± 1538	−3 (−31, +24)	0.80	−1 (−69, +66)	0.97
	Private label	49	777 ± 1137	773 ± 1135	−4 (−25, +15)	0.64		
Soup	Branded	90	299 ± 67	289 ± 68	−9 (−18, −1)	0.03	+3 (−14, +22)	0.65
	Private label	22	279 ± 64	273 ± 59	−6 (−16, +5)	0.26		
Vegetables	Branded	286	366 ± 465	366 ± 460	0 (−5, +5)	0.95	−7 (−17, +4)	0.24
	Private label	125	335 ± 424	328 ± 426	−7 (−17, +4)	0.23		

^1^ *p*-Value derived from paired *t*-tests, *p* ≤ 0.05 for a difference in sodium content between 2013 and 2011 for each of private label and branded grouped products; ^2^ *p*-Value derived from unpaired *t*-test, *p* ≤ 0.05 for a difference between the change in private label and branded in the rate of reformulation.

### 3.5. Differences in Mean Sodium for Products First Available in 2013

There were 1870 products available for the first time in 2013, 33% of which (*n* = 622) were private-label (Table 3). The overall mean sodium content of private-label products was −11% lower than that of branded products (−55, −100 to −9 mg/100 g; *p* = 0.02). When assessed by food category, the mean sodium content of new private-label breads and processed meats was 12% and 27% lower than the corresponding new branded products (both *p* ≤ 0.01). In contrast, new private-label sauce products contained on average 53% more sodium (*p* = 0.05) than their branded counterparts.

### 3.6. Change in Mean Sodium Content of Private-Label Products for Major Australian Retailers

**Figure 2:**
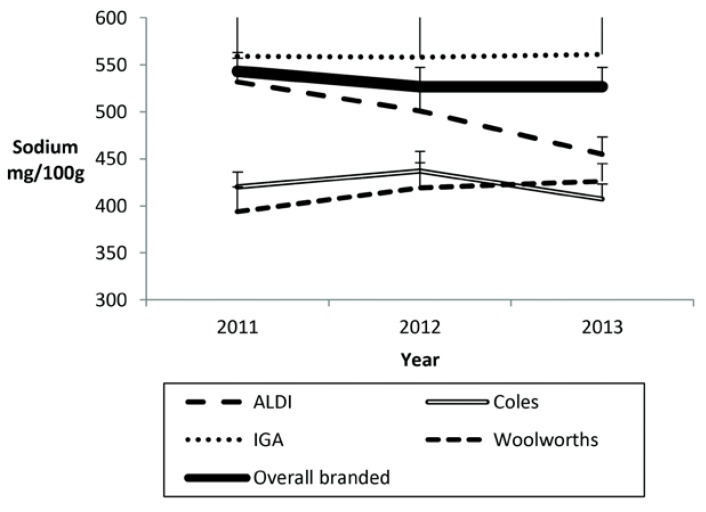
Mean sodium content (mg/100 g) and SE of supermarket private label products for supermarket retailers *versus* total branded (2011–2013).

Mean sodium content for Coles and for Woolworths products were lower than branded products in all years by between 17% and 27% (all *p* ≤ 0.002, Figure 2). Mean sodium content fell by 14% for ALDI private-label products between 2011 and 2013 (−77, −136 to −18 mg/100 g; *p* = 0.01) and in 2013 were 14% lower than branded products (*p* = 0.01) and not different from the 2013 mean content for Woolworths but still higher than Coles (+47, +1 to +95 mg/100 g; *p* = 0.04). The sodium content of products at IGA/Metcash were numerically higher than all others in all years with no evidence of improvement between 2011 and 2013 (*p* = 0.98).

**Table 3:** Mean sodium levels (mg/100 g) in supermarket private label and branded products new to the supermarket shelves in 2013 and the differences between them, overall and for 15 major food categories.

Food Category	Supply Type	*n* (%)	Mean Sodium mg/100 g ± SD	Mean Difference (Private Label—Branded) mg/100 g (95% CI)	*p*-Value ^1^
All products	Branded	1248 (67)	495 ± 516	−55 (−100, −9)	0.02
	Private label	622 (33)	440 ± 447		
Biscuits	Branded	163 (67)	411 ± 322	−59 (−129, +12)	0.10
	Private label	80 (33)	353 ± 227		
Bread	Branded	44 (50)	434 ± 113	−53 (−91, −14)	<0.01
	Private label	44 (50)	381 ± 59		
Breakfast cereals	Branded	54 (62)	136 ± 166	+43 (−35, +121)	0.28
	Private label	33 (38)	179 ± 195		
Cakes, muffins, pastries	Branded	75 (42)	245 ± 126	+43 (−2, +87)	0.06
	Private label	102 (58)	287 ± 162		
Cereal bars	Branded	54 (74)	128 ± 95	+26 (−26, +78)	0.32
	Private label	19 (26)	154 ± 107		
Cheese	Branded	107 (69)	671 ± 314	−52 (−152, +48)	0.30
	Private label	48 (31)	619 ± 230		
Crisps and snacks	Branded	66 (64)	660 ± 454	+47 (−119, +213)	0.58
	Private label	37 (36)	707 ± 308		
Desserts	Branded	28 (76)	111 ± 123	−8 (−95, +78)	0.85
	Private label	9 (24)	102 ± 59		
Nuts and seeds	Branded	48 (72)	129 ± 247	+46 (−99, +192)	0.53
	Private label	19 (28)	176 ± 317		
Processed fish	Branded	81 (78)	381 ± 177	−39 (−122, +44)	0.36
	Private label	23 (22)	342 ± 182		
Processed meat	Branded	140 (65)	1146 ± 471	−313 (−424, −202)	<0.001
	Private label	75 (35)	833 ± 347		
Ready meals	Branded	80 (65)	312 ± 185	+7 (−53, +67)	0.82
	Private label	44 (35)	319 ± 108		
Sauces	Branded	121 (83)	863 ± 934	+456 (−1, +912)	0.05
	Private label	24 (17)	1320 ± 1219		
Soup	Branded	73 (72)	268 ± 64	+2, (−26, +28)	0.93
	Private label	29 (28)	270 ± 61		
Vegetables	Branded	114 (76)	300 ± 417	−110 (−257, +37)	0.14
	Private label	36 (24)	190 ± 275		

^1^ *p*-Value derived from unpaired *t*-tests, *p* ≤ 0.05 for a difference in sodium content between private labels and branded grouped products.

## 4. Discussion

Private-label products are traditionally perceived as low-cost, low-quality choices when compared to their branded counterparts. Recent data report although perceptions of private-label quality have improved Australian consumers still have quality concerns [18]. These data suggest the quality concerns are unfounded in at least one important regard—the overall mean sodium content of Australian private-label products was consistently and substantially lower than that of branded products for the three years from 2011 to 2013. Furthermore, the difference was substantial at 17% less sodium. A lower sodium intake across the Australian population would translate into thousands less heart attacks and strokes each year and hundreds of millions of dollars savings through health care costs avoided [9]. Excess sodium intake is a primary cause of high blood pressure which is a leading cause of premature death and disability in Australia and most other countries around the world.

The lower mean sodium content in private-label compared to branded products was a consequence of numerically lower mean sodium content for 11 of the 15 food categories studied. These differences were statistically significantly for four of these categories. Of which two (bread and processed meats) were prioritised for salt reduction by the FHD based on their relative high contribution to dietary intake of sodium [24]. The corresponding numbers for branded products were four categories with numerically lower mean sodium content and just one, breakfast cereals also prioritised by the FHD for salt reduction, with a statistically significantly lower value. These differences were mostly already apparent in 2011 and persisted through to 2013 with little evidence of a change in the pattern over these three years—there were very small and comparable reductions in mean sodium content attributable to reformulation for both private-label and branded products, but private-label continued to introduce to the market products that were on average lower in sodium than new branded products. The reason why private-label products already had sodium content so much lower than branded products in 2011 is unclear but the production of foods lower in sodium by the major retailers seems likely to be a long-standing phenomenon.

There was also marked difference in the mean sodium content of the private-label products provided by different retailers. IGA (Metcash) stood out as having consistently higher mean sodium content than its counterparts, and ALDI for the very encouraging reduction in mean sodium content across its private-label range. ALDI aside, the absence of any overall change in mean sodium content for either private-label or branded products grouped, or by any of the other retailers, suggests limited activity on sodium reduction by most food suppliers across most food categories.

Our study data included well-known brand names, supplied by large manufacturers, many of whom have publicly committed to producing healthier foods [24,29]. Assessing individual manufacturers was beyond the scope of our study, but it is probable that some companies have reduced the average sodium content of their product portfolio while others have not. Prior studies have also shown substantial heterogeneity in sodium reduction between branded food manufacturers, [25,30,31,32,33] and our finding highlight such variability is also present amongst private-label. Government leadership of the FHD can establish a level playing field for sodium reduction in a broad range of food categories for supermarket retailers and branded manufacturers to work towards. A broadening of the FHD targets and strengthening private sector accountability will be vital if Australia is to have any chance of delivering upon the sodium reduction target committed to as part of the World Health Organization 25 by 25 chronic disease prevention goal [34].

A key strength of our study is the consistent and systematic sampling method used to collect data over the years. Longitudinal data has allowed us to assess the overall sodium content of foods across time while separately evaluating the contribution that reformulation of established products and the development of new lower salt products has made. The large number of products sampled provided good coverage of foods in each category and enhanced statistical power to assess differences in mean sodium content. The inclusion of a broad range of categories makes an important contribution to understanding the status of sodium reduction across most of the packaged, processed food supply. The detailed categorisation of products enabled us to explore the product mix of private-label *versus* branded products. Including product sub-categories with extreme values of products eaten infrequently did not substantially alter the findings but in the case of sauces highlighted the importance of knowing product mix to interpreting the study findings.

The data also have some limitations and are likely to be incomplete because they were collected from only one store location for each of the four supermarkets. Incompleteness may be greater for private-label than branded products since branded products could be marketed by multiple different retailers but private-label products by only the parent retailer. We were also unable to include foods for sale exempt from labelling requirements such as products made and packaged on the premises from which it is sold or packaged and displayed in an assisted service display cabinet [35]. We relied on the validity of the nutritional information on the NIP, although prior studies suggest this is generally accurate and reliable [36]. In the absence of an agreed definition of “premium” and way to categorise private-label brands we were unable to differentiate between the tiered private-label brands that some retailers now market as value-based, mid-range, and premium [16]. We also did not examine price and as our study examined foods only from Australia, which is unique in both the level of supermarket concentration and private-label share [16], our results may not be generalizable to other countries.

## 5. Conclusions

In conclusion, private-label products performed generally better than branded in terms of sodium content. This is promising news for public health since lower income families suffer the greatest burden of blood pressure-related disease and are more likely to purchase private-label products [16,20,37]. Even a modestly lower sodium content, if delivered across a broad range of product categories and food suppliers, could produce large overall health gains [8]. A better understanding of why the supermarkets provide lower sodium foods would be of great value because supermarket ranging decisions have a profound impact upon the availability, accessibility, affordability and acceptability of foods. Identifying the mechanism by which the lower sodium content of private-label products has been achieved and extending this to branded products should be a priority.

## Supplementary Files

Supplementary File 1
