# Supplementary material for: A Comparison of the Sodium Content of Supermarket Private-Label and Branded Foods in Australia
Source: Nutrients. 2015 Aug 21;7(8):7027–41. doi: 10.3390/nu7085321 (PMC4555160; doi:10.3390/nu7085321)
Supplement: Supplementary File 1 [file nutrients-07-05321-s001.docx]

Supplementary

**Table S1.** Major food categories and sub-categories included in analyses, and the number and proportion of private-label products (2013) expressed as percentage of all private-label products, and all products within a category.

| **Major Food Category** | **Foods Included** | **The Number and Proportion of  Private-Label Products Expressed as a Percentage of All Private-Label Products (*n* = 1849)  in 2013** | **The Number and Proportion of  Private-Label Products Expressed as a Percentage of the Total Number of Products (Private-Label plus Branded) for Each Category in 2013** |
| --- | --- | --- | --- |
| Biscuits | Anzac style biscuits; Breadsticks;  Choc-chip biscuits; Chocolate coated biscuits; Chocolate-covered sandwich style biscuits  (e.g., Tim Tam); Crackers (e.g., water crackers); Crispbread (e.g., Salada); Flavoured crackers and crispbread; Flavoured rice crackers; Fruit and nut chunks biscuits; Fruit filled biscuits; Gingerbread biscuits; Iced biscuits; Jam filled biscuits; Other sweet filled biscuits; Other sweet unfilled biscuits; Plain sweet unfilled biscuits; Rice cakes and similar products, plain; Rice crackers, plain; Shortbread style biscuits; Wafer-style filled biscuit | 214/1849 (12%) | 214/845 (25%) |
| Bread | Bread rolls; Crumpets; English muffins; Garlic bread; Multigrain bread; Other plain bread; Rye bread; Soy & linseed bread; White bread; Wholemeal bread | 99/1849 (5%) | 99/277 (36%) |
| Breakfast cereals | Breakfast biscuits; Cocoa pop style cereal; Corn flakes; Flakes with additions; Granola/cluster cereals; Muesli with fruit; Nutri-Grain style cereal; Other plain flakes; Other sweet style cereal; Plain brans; Plain muesli; Rice bubbles | 85/1849 (5%) | 85/276 (31%) |
| Cakes, muffins and pastries | Brownie mixes; Carrot cakes; Chocolate cake mixes; Chocolate cakes; Chocolate/choc-chip muffins; Cupcake mixes; Cupcakes; Filo/frozen pastry; Fruit cakes; Fruit-based muffins; Lamingtons; Muffin mixes; Pancake mixes; Plain cake mixes; Plain cakes; Slices; Sweet pastries (e.g., tarts); Swiss rolls | 196/1849 (11%) | 196/377 (52%) |
| Cereal bars | Baked/filled bars; Chocolate-coated cereal-based bars; Plain cereal-based bars; Puff-based bars; Yoghurt-coated cereal-based bars | 46/1849 (2%) | 46/183 (25%) |

**Table S1.** *Cont.*

| **Major Food Category** | **Foods Included** | **The Number and Proportion of  Private-Label Products Expressed as a Percentage of All Private-Label Products (*n* = 1849)  in 2013** | **The Number and Proportion of  Private-Label Products Expressed as a Percentage of the Total Number of Products (Private-Label plus Branded) for Each Category in 2013** |
| --- | --- | --- | --- |
| Cheese | Block hard cheeses; Blue cheese; Bocconcini; Camembert and brie; Cottage cheese; Cream cheese; Feta; Flavoured cream cheese; Grated cheese; Haloumi; Parmesan; Processed cheese; Ricotta; Sliced cheese | 145/1849 (8%) | 145/538 (27%) |
| Crisps and snacks | BBQ flavour potato crisps; Cheese-based snack packs; Chicken flavour potato crisps; Corn chips; Extruded snacks; Fish-based snack packs; Other flavours potato crisps not otherwise specified; Plain potato crisps; Popcorn; Salt and vinegar potato crisps; Sour cream and chives potato crisps; Sweet chilli potato crisps; Wholegrain chips | 109/1849 (6%) | 109/309 (35%) |
| Desserts | Cheesecake; Custard; Mousses; Other prepared desserts not otherwise specified; Puddings;  Rice puddings | 36/1849 (2%) | 36/132 (27%) |
| Nuts and seeds | Almonds; Brazil nuts; Cashews; Macadamias; Mixed nuts; Peanuts; Pine nuts; Pistachios;  Seeds; Walnuts | 120/1849 (6%) | 120/318 (38%) |
| Processed fish | Canned shellfish; Coated frozen fish; Flavoured canned salmon; Flavoured canned sardines; Flavoured canned tuna; Other canned fish; Plain canned salmon; Plain canned sardines; Plain canned tuna in oil; Plain canned tuna in water; Uncoated frozen fish | 169/1849 (9%) | 169/456 (37%) |
| Processed meat | Bacon; Beef burgers; Beef sausages; Cabanossi and twiggy sticks; Canned beef; Canned chicken; Canned ham (e.g., SPAM); Chorizo; Hotdogs; Kransky; Lamb sausages; Pancetta and prosciutto; Pork sausages; Salami; Sausage rolls; Sliced beef; Sliced chicken; Sliced ham; Sliced luncheon meat; Sliced turkey; Whole hams and similar products | 179/1849 (10%) | 179/515 (35%) |

**Table S1.** *Cont.*

| **Major Food Category** | **Foods Included** | **The Number and Proportion of  Private-Label Products Expressed as a Percentage of All Private-Label Products (*n* = 1849)  in 2013** | **The Number and Proportion of  Private-Label Products Expressed as a Percentage of the Total Number of Products (Private-Label plus Branded) for Each Category in 2013** |
| --- | --- | --- | --- |
| Ready meals | Chilled Asian-style meals; Chilled meat-based lasagne; Chilled meat-based pasta meals; Chilled vegetarian pasta-based meals; Frozen Asian-style meals; Frozen meat-based lasagne; Frozen  meat-based pasta meals; Frozen risotto; Frozen vegetarian lasagne; Frozen vegetarian  savoury-filled foods (e.g., samosas) | 84/1849 (5%) | 84/256 (33%) |
| Sauces | Asian-style liquid recipe bases; BBQ sauce;  Cheese sauce mixes; Chilli and sweet chilli sauces; Cream-based fresh pasta sauces; Dijon mustard; Fresh pasta sauce with meat; Gravy as prepared; Hot English mustard; Pesto; Seeded mustard; Soy sauce; Stock as prepared; Sweet and sour ambient meal-based sauces; Tomato paste; Tomato sauce; Tomato-based ambient pasta sauces; Tomato-based fresh pasta sauces | 100/1849 (5%) | 100/589 (17%) |
| Soups | Canned chicken and corn soup; Canned  chicken-based soup; Canned minestrone soup; Canned other soup with meat; Canned other soup with veg; Canned pumpkin soup; Canned tomato soup; Chicken and corn dry soup mix;  Chicken-based dry soup mix; Chilled minestrone soup; Chilled pumpkin soup; Other soup with veg dry mix; Pumpkin dry soup mix; Soup with pasta/noodles dry mix; Tomato dry soup mix | 60/1849 (3%) | 60/253 (24%) |
| Vegetables | Artichokes; Baked beans; Bean mixes; Butter beans; Canned asparagus; Canned beetroot; Canned corn; Canned mushrooms; Canned tomatoes; Cannellini beans; Chick peas; Chilli beans; Dolmades; Hash browns and other frozen potato products; Kidney beans; Lentils; Olives; Onions; Other canned vegetables not otherwise specified; Peas; Pickles/gherkins; Plain frozen vegetables; Potato chips; Potato wedges; Sundried tomatoes | 207/1849 (11%) | 207/674 (31%) |

**Table S2.** Mean **s**odium levels (mg/100 g) in all private-label and branded products on the supermarket shelves in 2011 and 2013 and the change between 2011 and 2013, overall and for 15 major food categories.

|  | **Supply Type** | **Year** | | | | **Mean Change (2013–2011) within Private-Label or Branded** | |
| --- | --- | --- | --- | --- | --- | --- | --- |
|  |  | **2011** | | **2013** | |  |  |
|  |  | ***n*** | **Mean Sodium mg/100 g ± SD** | ***n*** | **Mean Sodium mg/100 g ± SD** | **Mean Change mg/100 g (95% CI)** | ***p*-Value ^1^** |
| All products | Branded | 3067 | 543 ± 690 | 4146 | 527 ± 655 | −16 (−47, +16) | 0.34 |
|  | Private label | 1434 | 463 ± 517 | 1849 | 437 ± 454 | −26 (−60, +8) | 0.13 |
| Biscuits | Branded | 356 | 453 ± 330 | 631 | 450 ± 344 | −43 (−48, +39) | 0.85 |
|  | Private label | 164 | 339 ± 254 | 214 | 340 ± 242 | +1 (−50, +52) | 0.96 |
| Bread | Branded | 128 | 482 ± 97 | 178 | 453 ± 100 | −29 (−51, −6) | 0.01 |
|  | Private label | 61 | 463 ± 88 | 99 | 421 ± 85 | −42 (−70, −14) | <0.001 |
| Breakfast cereals | Branded | 141 | 197 ± 184 | 191 | 144 ± 160 | −53 (−91, −15) | 0.01 |
|  | Private label | 32 | 162 ± 189 | 85 | 197 ± 193 | +35 (−45, +113) | 0.39 |
| Cakes, muffins, pastries | Branded | 108 | 335 ± 131 | 181 | 308 ± 138 | −27 (−59, +5) | 0.09 |
|  | Private label | 108 | 293 ± 113 | 196 | 291 ± 145 | −2 (−31, +28) | 0.92 |
| Cereal bars | Branded | 74 | 163 ± 105 | 137 | 137 ± 101 | −26 (−57, +4) | 0.09 |
|  | Private label | 28 | 130 ± 98 | 46 | 151 ± 110 | +21 (−29, +70) | 0.40 |
| Cheese | Branded | 306 | 754 ± 379 | 393 | 752 ± 371 | −2 (−58, +55) | 0.96 |
|  | Private label | 115 | 699 ± 366 | 145 | 703 ± 372 | +4 (−87, +94) | 0.93 |
| Crisps and snacks | Branded | 180 | 687 ± 351 | 200 | 664 ± 419 | −23 (−101, +54) | 0.56 |
|  | Private label | 84 | 647 ± 332 | 109 | 678 ± 326 | +31 (−63, +125) | 0.52 |
| Desserts | Branded | 92 | 83 ± 70 | 96 | 113 ±109 | +30 (+4, +56) | 0.03 |
|  | Private label | 26 | 94 ± 80 | 36 | 83 ± 55 | −11 (−47, +26) | 0.56 |
| Nuts and seeds | Branded | 163 | 109 ± 244 | 198 | 118 ± 238 | +9 (−41, +59) | 0.73 |
|  | Private label | 111 | 182 ± 233 | 120 | 140 ± 234 | −42 (−102, +18) | 0.18 |
| Processed fish | Branded | 256 | 399 ± 154 | 287 | 395 ± 149 | −4 (−30, +21) | 0.74 |
|  | Private label | 169 | 392 ± 152 | 169 | 371 ± 152 | −21 (−54, +11) | 0.19 |
| Processed meats | Branded | 232 | 1068 ± 517 | 336 | 1095 ± 491 | +27 (−58, +111) | 0.54 |
|  | Private label | 146 | 957 ± 419 | 179 | 850 ± 375 | −107 (−194, −19) | 0.02 |
| Ready meals | Branded | 94 | 270 ± 65 | 172 | 295 ± 140 | +25 (0, +50) | 0.05 |
|  | Private label | 42 | 268 ± 73 | 84 | 318 ± 108 | +50 (+18, +82) | <0.01 |
| Sauces | Branded | 397 | 1052 ± 1492 | 489 | 1032 ± 1430 | −20 (−214, +174) | 0.84 |
|  | Private label | 100 | 889 ± 1219 | 100 | 950 ± 1219 | +61 (−298, +420) | 0.74 |
| Soup | Branded | 145 | 299 ± 79 | 193 | 281 ± 68 | −18 (−33, −1) | 0.04 |
|  | Private label | 45 | 290 ± 71 | 60 | 271 ± 63 | −19 (−46, +8) | 0.16 |
| Vegetables | Branded | 395 | 378 ± 463 | 464 | 359 ± 456 | −19 (−81, +43) | 0.55 |
|  | Private label | 203 | 309 ± 403 | 207 | 308 ± 408 | −1 (−80, +77) | 0.97 |

^1^ *p*-Value derived from unpaired *t* tests, *p* ≤ 0.05 for a difference in sodium content between private-label and branded
grouped products.

**Table S3.** Mean **s**odium levels (mg/100 g) in private-label and branded products on the supermarket shelves in 2013 and the differences between, overall and for 15 food major categories without removing 186 products present in 5 sub-categories.

| **Food Category** | **Supply Type** | ***n* (%)** | **Mean Sodium mg/100 g ± SD** | **Mean Difference (Private Label—Branded) mg/100 g (95% CI)** | ***p*-Value ^1^** |
| --- | --- | --- | --- | --- | --- |
| All products | Branded | 4315 (70) | 596 ± 844 | −156 (−188, −123) | <0.001 |
|  | Private label | 1866 (30) | 440 ± 457 |  |  |
| Biscuits | Branded | 631 (75) | 450 ± 344 | −110 (−151, −67) | <0.001 |
|  | Private label | 214 (25) | 340 ± 242 |  |  |
| Bread | Branded | 178 (64) | 453 ± 100 | −32 (−56, −9) | 0.01 |
|  | Private label | 99 (36) | 421 ± 85 |  |  |
| Breakfast cereals | Branded | 191 (69) | 144 ± 160 | +53 (+4, +100) | 0.03 |
|  | Private label | 85 (31) | 197 ± 193 |  |  |
| Cakes, muffins, pastries | Branded | 181 (48) | 308 ± 138 | −17 (−45, +12) | 0.26 |
|  | Private label | 196 (52) | 291 ± 145 |  |  |
| Cereal bars | Branded | 137 (75) | 137 ± 101 | +14 (−20, +49) | 0.43 |
|  | Private label | 46 (25) | 151 ± 110 |  |  |
| Cheese | Branded | 393 (73) | 752 ± 371 | −49 (−120, +22) | 0.17 |
|  | Private label | 145 (27) | 703 ± 372 |  |  |
| Crisps and snacks | Branded | 200 (65) | 664 ± 419 | +14 (−77, +105) | 0.76 |
|  | Private label | 109 (35) | 678 ± 326 |  |  |
| Desserts | Branded | 96 (73) | 113 ±109 | −30 (−58, −1) | 0.04 |
|  | Private label | 36 (27) | 83 ± 55 |  |  |
| Nuts and seeds | Branded | 198 (62) | 118 ± 238 | +22 (−32, +76) | 0.42 |
|  | Private label | 120 (38) | 140 ± 234 |  |  |
| Processed fish ^2^ | Branded | 296 (64) | 415 ± 217 | −39 (−76, −2) | 0.04 |
|  | Private label | 173 (36) | 376 ± 155 |  |  |
| Processed meat | Branded | 336 (65) | 1095 ± 491 | −245 (−321, −168) | <0.001 |
|  | Private label | 179 (35) | 850 ± 375 |  |  |
| Ready meals | Branded | 271 (67) | 295 ± 140 | +23 (−11, +58) | 0.18 |
|  | Private label | 117 (33) | 318 ± 108 |  |  |
| Sauces ^2^ | Branded | 598 (85) | 1339 ± 1678 | −372 (−640, −105) | 0.01 |
|  | Private label | 105 (15) | 967 ± 1200 |  |  |
| Soup | Branded | 193 (76) | 281 ± 68 | −10 (−29, +10) | 0.31 |
|  | Private label | 60 (24) | 271 ± 63 |  |  |
| Vegetables ^2^ | Branded | 515 (71) | 475 ± 950 | −155 (−254, −54) | 0.002 |
|  | Private label | 215 (29) | 320 ± 421 |  |  |

^1^ *p-*Value derived from unpaired *t* tests, *p* ≤ 0.05 for a difference in sodium content between private-label and branded grouped products; ^2^ Products with extreme sodium values were excluded from these categories in the primary analyses, but in this table they are included.
